# 

**Published:** 1878-04

**Authors:** 


### CONTENTS.

COMMUNICATIONS.

Annual Address.—*By A. O. Rawles, D. D. S.* ........ 133

Practical Dentistry.—*By W. C. Duncan, D. D S.* ........ 149

PROCEEDINGS OF SOCIETIES.

Discussions before the Mississippi Valley Dental Association........ 152

EDITORIAL.

Commencement........ 171
Commencement........ 172
History of Dentistry........ 172
California State Dental Association........ 173
Illinois State Dental Society........ 174
Eastern Indiana Dental Association........ 175
Mississippi Valley Dental Association........ 176

